# VP Shunt Malfunction Simulation Case for Emergency Medicine Residents

**DOI:** 10.5070/M5.52255

**Published:** 2026-07-31

**Authors:** Alexis Koda, Kyle Thomas Vawter

**Affiliations:** * University of California, Irvine, Department of Emergency Medicine, Orange, CA; ^ Lehigh Valley Health Network, Department of Emergency Medicine, Allentown, PA

## Abstract

**Audience:**

This simulation was designed primarily for emergency medicine residents and pediatric emergency medicine residents. Additionally, off-service residents, neurosurgical residents, pediatric residents, faculty, and medical students may benefit.

**Introduction:**

Ventriculoperitoneal (VP) shunts are the treatment of choice for patients suffering from hydrocephalus. Hydrocephalus affects about 125,000 patients in the United States, and 33,000 VP shunts are implanted in patients every year.^1^ However, these devices can malfunction and can cause complications. It is important for the emergency department physician to recognize when these devices are not functioning correctly to prevent life-threatening complications. Training for VP shunt malfunction can be variable depending upon the patient population seen by residents. Therefore, we outline a simulation for mechanical VP shunt malfunction to provide learners with exposure to this topic.

**Educational Objectives:**

The objective of this simulated case is to increase learner knowledge and develop the skills necessary to troubleshoot VP shunt malfunction. By the end of this simulation, learners should be able to: 1) review pertinent historical and physical exam points for a shunt patient, 2) recognize the signs and symptoms of VP shunt malfunction, 3) review an algorithm for diagnosing VP shunt malfunction, 4) discuss treatment for common complications of VP shunt dysfunction, and 5) recall the indications for an emergent VP shunt tap.

**Educational Methods:**

In this study, a high-fidelity mannikin with a do-it-yourself (DIY) task trainer was used in a simulated case scenario. Learners were required to recognize the signs and symptoms of shunt dysfunction, administer the appropriate treatment, and appropriately disposition the simulated patient.

**Research methods:**

Residents completed pre- and post-simulation surveys to assess their perceived knowledge and confidence regarding the diagnosis and management of VP shunt malfunction. To prevent priming or diagnostic cueing, the term “neurologic device” was used in the pre-simulation surveys. This wording may significantly limit direct comparison between pre- and post-simulation responses. Learners were also asked to provide feedback on the utility of the simulation for their future practice and usefulness of the topic.

**Results:**

Learners reported high levels of confidence in recognizing signs and symptoms of shunt dysfunction and managing complications after the simulation. Residents also reported that the case was valuable for their future clinical practice and felt as though the simulation experience was positive.

**Discussion:**

Overall, the educational content was effective. The simulation appears to be useful in addressing knowledge gaps in managing VP shunt dysfunction and may increase learner confidence.

**Topics:**

Medical simulation, ventriculoperitoneal shunt dysfunction, hydrocephalus, pediatrics, increased intracranial pressure, shunt tap, neurologic emergencies.

## USER GUIDE

**Table:** 

List of Resources:	
Abstract	1
User Guide	3
Instructor Materials	7
Operator Materials	16
Debriefing and Evaluation Pearls	20
Simulation Assessment	24
Appendix A: VP Shunt Dysfunction PowerPoint	29

**Learner Audience:**

This exercise is directed primarily toward junior and senior residents. This case may be challenging for interns.

However, the material is still useful for them, and they may still benefit in participating in a team approach. The material is more appropriate for senior residents, pediatric fellows, and faculty.

**Time Required for Implementation:**

**Preparation:** 30 minutes (15 minutes task trainer creation +15 minutes mannikin programming)

**Time for case:** 20 minutes

**Time for debriefing:** 20–30 minutes

**Recommended Number of Learners per Instructor:**

3–5 learners

**Topics:**

Medical simulation, ventriculoperitoneal shunt dysfunction, hydrocephalus, pediatrics, increased intracranial pressure, shunt tap, neurologic emergencies.

**Objectives:**

By the end of this simulation, learners should be able to:

1. Review pertinent historical and physical exam points for a shunt patient
2. Recognize the signs and symptoms of VP shunt malfunction
3. Review an algorithm for diagnosing VP shunt malfunction
4. Discuss treatment for common complications of VP shunt dysfunction
5. Recall the indications for an emergent VP shunt tap.

### Linked objectives and methods

The goals and objectives of this simulation are achieved through a structured simulated experience that utilizes Kolb’s experiential learning theory. This simulation allows residents to practice a focused history and exam pertinent to the VP shunt patient and recognize signs and symptoms of shunt dysfunction (Objectives 1 & 2). Residents must develop a broad differential and adopt a systematic approach to identifying problems with the shunt (Objective 3). As the case unfolds, learners must identify the cause of the shunt dysfunction and recognize that early neurosurgical input is required (Objective 4). They must also recognize signs of increased intracranial pressure. Residents should also be familiar with performing the shunt tap as a procedure to be done sparingly with direction from neurosurgery unless life-threatening increased intracranial pressure (ICP) complications occur (Objective 5). The simulation provided learners with an opportunity to apply medical knowledge, clinical reasoning, and communication skills through scenario participation and facilitated debriefing.

The structured debrief following the scenario reinforces these objectives as well as allowing for debriefing on communication skills. Learners were also expected to communicate clearly and develop team leadership skills using closed loop communication with team members. They also needed to communicate findings clearly in lay terms to the mother about what was going on with the patient.

### Recommended pre-reading for instructor

We recommend that instructors review literature involving VP shunt dysfunction including diagnosis, signs and symptoms, and management. Suggested readings are included below.^2^–^6^

- Fox SM. Tapping a VP Shunt. Pediatric EM Morsels. September 16, 2011. Accessed December 7, 2024. https://pedemmorsels.com/tapping-a-vp-shunt/
- VP Shunt. Published online June 2018. Accessed March 5, 2025. https://www.emrap.org/episode/thebrain/vpshunt
- Kim TY, Stewart G, Voth M, Moynihan JA, Brown L. Signs and symptoms of cerebrospinal fluid shunt malfunction in the pediatric emergency department. *Pediatr Emerg Care*. 2006;22(1):28–34. doi:10.1097/01.pec.0000195764.50565.8c
- Conforto A, Wagner JG. Management of increased intracranial pressure and intracranial shunts. In: Roberts JR, ed. *Roberts and Hedges’ Clinical Procedures in Emergency Medicine and Acute Care*. 8th ed. Elsevier; 2019:1259–1274.
- Bober J, Rochlin J, Marneni S. Ventriculoperitoneal shunt complications in children: An evidence-based approach to emergency department management. *Pediatr Emerg Med Pract*. Published online February 2, 2016. https://www.ebmedicine.net/topics/neurologic/pediatric-ventriculoperitoneal-shunt

### Shunt Model Innovation

List of items required to replicate this innovation**:**[Fig f1-jetem-11-3-s1]

**Figure 1 f1-jetem-11-3-s1:**
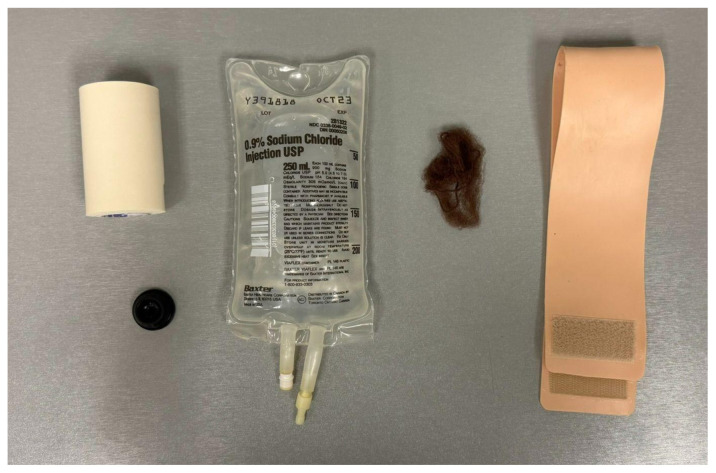
Materials needed

1. foam tape (hospital supply or Amazon)
2. 250 mL saline bag (hospital supply)
3. spare IV bag injection port or rubber stopper from medication vial (hospital supply)
4. (optional) artificial skin (Amazon)
5. (optional, if mannikin has hair) wig cap or stockinette (Amazon or hospital supply)

Detailed methods to construct this innovation**:**

1. Remove air and enough fluid to roll the 250 mL saline bag to approximately 1” wide. Make sure that the bag is tense enough to palpate.[Fig f2-jetem-11-3-s1]
  **Figure 2 f2-jetem-11-3-s1:**
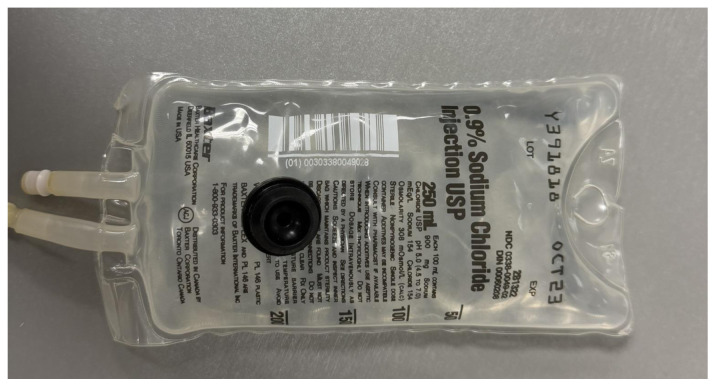
Step 2 of assembly. Note that the bag should be rolled up.
2. Cut out spare IV bag injection port or remove rubber stopper from medication vial and place on saline bag.
3. Secure rubber piece to the bag with foam tape (this can serve as skin layer or may attach artificial skin layer on top).[Fig f3-jetem-11-3-s1]
  **Figure 3 f3-jetem-11-3-s1:**
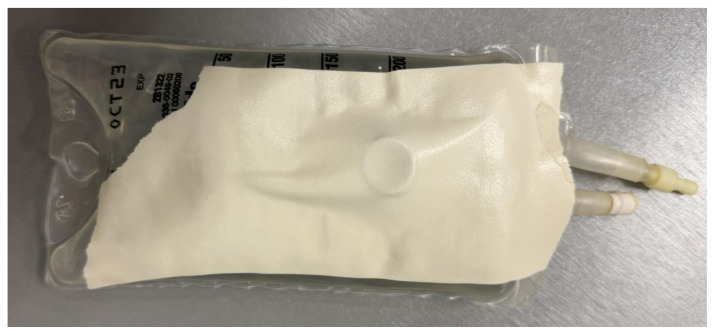
Step 3 of Assembly
4. Tape to skin on the right parietal scalp of the mannikin. Secure with hair net or wig cap if there is hair.[Fig f4-jetem-11-3-s1][Fig f5-jetem-11-3-s1][Fig f6-jetem-11-3-s1]
  **Figure 4 f4-jetem-11-3-s1:**
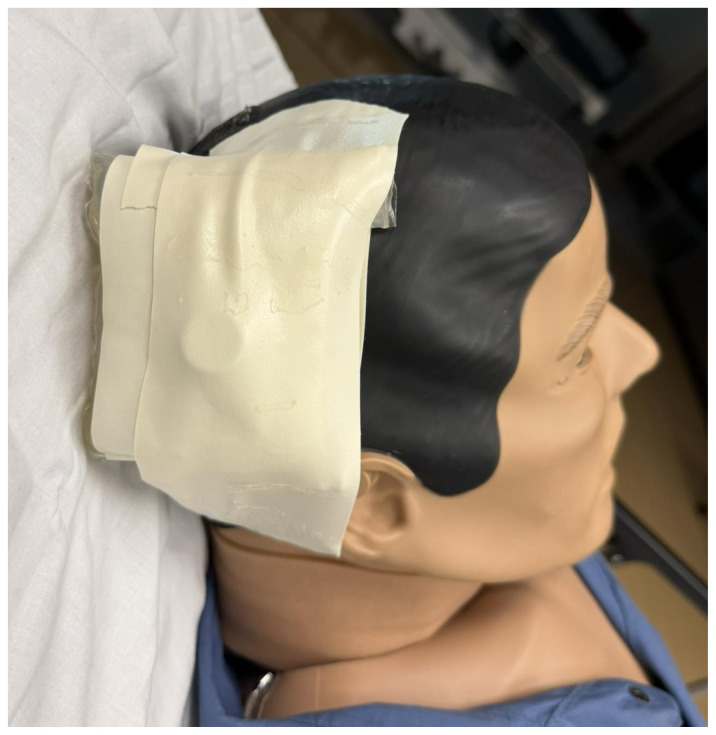
Step 4 of assembly

**Figure 5 f5-jetem-11-3-s1:**
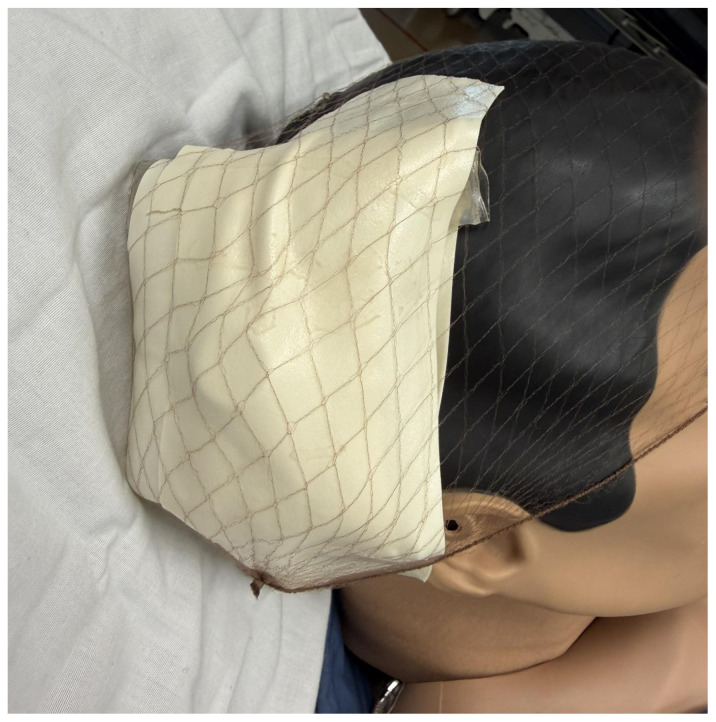
Modification for mannikins with hair. May attach the model to the mannikin with a wig cap or hair net.

**Figure 6 f6-jetem-11-3-s1:**
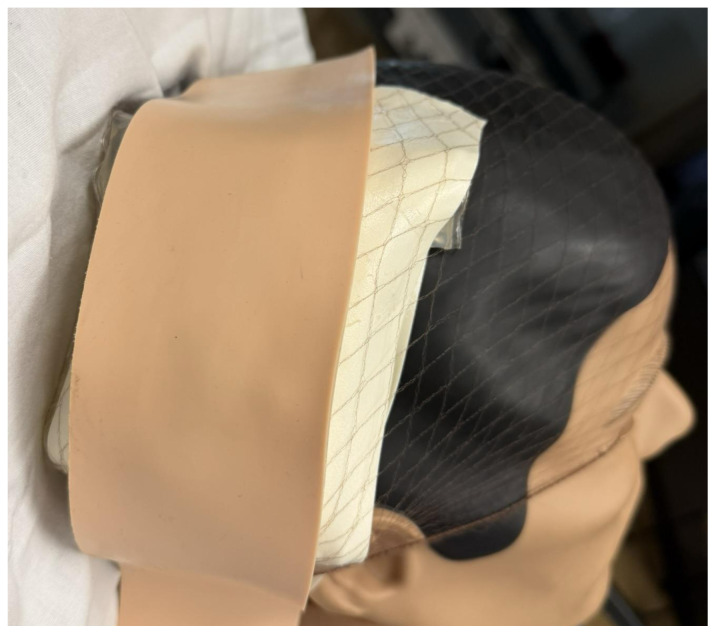
Optional step. May place artificial skin on top.

### Results and tips for successful implementation

This simulated case took place in a simulation room with a high fidelity mannikin. The scenario was run with one faculty member and two teams of residents that contained five and six members from all three years of a three-year residency program containing categorical EM residents only. Teams consisted of a team leader who was a second-year resident advised by a third-year resident. The second-year resident assigned other team roles. These were not ideal conditions. Ideally, the case would have been run with the help of at least one actor, and a smaller group would have been preferred. However, the group size was determined by logistical constraints and timed before and after the usual didactic schedule.

Overall, the results for the simulation were positive with the residents reporting high levels of comfort recognizing signs and symptoms and managing complications of VP shunt dysfunction. After the case, the residents reported that they did not have much previous experience with VP shunt dysfunction but felt that the simulation was helpful to their future practice in emergency medicine. Table 1 shows these results.

Based on implementation experience with this simulation, we identified several key refinements to enhance future iterations. The VP shunt reservoir model’s anatomical positioning was challenging due to constraints imposed by the mannequin’s hair, which limited realistic placement for examination. We also noted that learners frequently overlooked reassessment of neurological status prior to intubation, suggesting a need for more explicit prompting for this critical step. Furthermore, when learners proposed inappropriate decompressive procedures for this clinical scenario, we found that having an assistant available to provide subtle redirection improved the educational experience without disrupting case flow. Next, following the simulation, facilitated discussion about the appropriateness of CT imaging despite radiation concerns proved valuable in addressing common clinical hesitations observed during the case. Finally, it is important to emphasize when the shunt tap should be considered and that it should only be performed under the direction of a neurosurgeon. There were no modifications that we made as a result of the feedback; however, the scenario potentially could be modified for an oral boards style case or may be adapted to provide continuing education and retraining for faculty who may need a refresher on the material.

### Associated content

See PowerPoint debrief

## Supplementary Information





## Figures and Tables

**Table:** 

**#1**	**CBC**
**#2**	**CMP**
**#3**	**Coagulation Studies**
**#4**	**Abdominal X-Ray**
**#5**	**Head CT**
**#6**	**Post-intubation X-Ray**

**Table:** 

**Vitals:**	Heart Rate (HR) 60	Blood Pressure (BP) 160/60
	Respiratory Rate (RR) 22	Temperature (T) 37.0°C
	Oxygen Saturation (O_2_Sat) 100% on room air (RA)	

**Table:** 

*CBC (Complete Blood Count)*	
White blood count (WBC)	11.5 × 1000/mm^3^
Hemoglobin (Hgb)	15.4 g/dL
Hematocrit (HCT)	46.2%
Platelet (Plt)	250 × 1000/mm^3^

**Table:** 

*Comprehensive metabolic panel (BMP)*	
Sodium	145 mEq/L
Potassium	4.0 mEq/L
Chloride	110 mEq/L
Bicarbonate (HCO3)	24 mEq/L
Blood Urea Nitrogen (BUN)	25 mg/dL
Creatinine (Cr)	1.0 mg/dL
Glucose	110 mg/dL
Aspartate Amino Transferase (AST)	10 U/L
Alanine Amino Transferase (ALT)	10 U/L
Alkaline Phosphatase (Alk phos)	400 U/L

**Table:** 

*Coagulation Profile*	
Prothrombin Time (PT)	12.0 seconds
Partial Thromboplastin Time (PTT)	44.0 seconds
International Normalized Ratio (INR)	1.0

**Figure:**
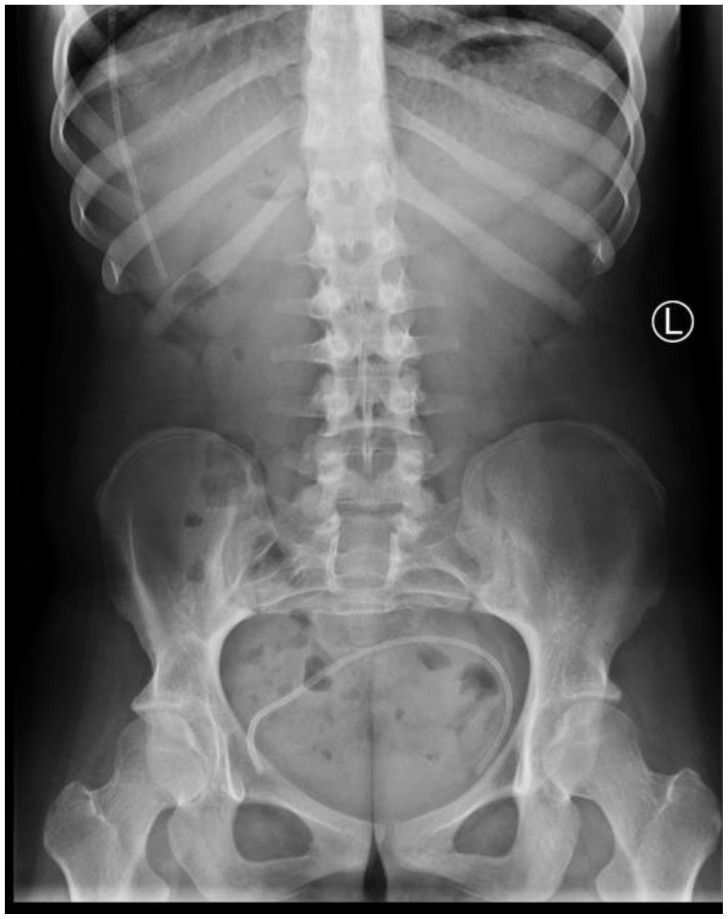


**Figure:**
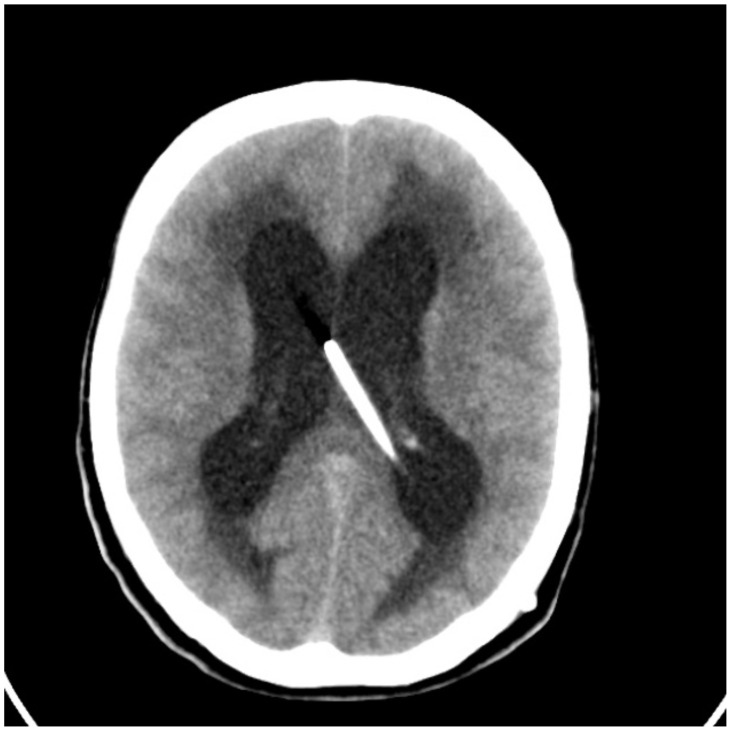


**Figure:**
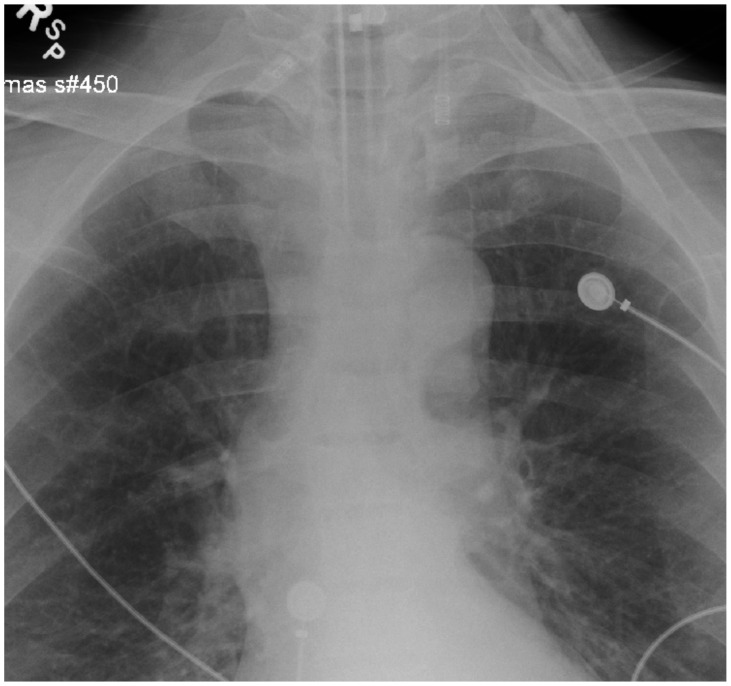


**Table:** SIMULATION EVENTS TABLE:

Minute (state)	Participant action/ trigger	Patient status (simulator response) & operator prompts	Monitor display (vital signs)
Baseline	Assess ABCs, obtain point-of-care glucose, and obtain focused history and physical. Order shunt series and infectious workup.	Patient is lying in the bed. Able to answer questions slowly. Glasgow Coma Scale 14: E-4 V-5 M-5. Moving all extremities. Follows commands. The patient states, “my tummy doesn’t feel good” and “I’m tired.” If no VP shunt series is ordered, the nurse can ask, “Doctor, what orders do you want?” “Do you want any imaging?”	T: 37.0° C HR: 80 BP: 127/86 RR: 18 O2: 100%
Results	Interpret results.	**Display shunt series** showing shunt fracture in the abdomen and **CT head** showing ventriculomegaly. Display **labs.** If no interpretation is provided, mother can ask, “what do the test results show?”	T: 37.0° C HR: 80 BP: 127/86 RR: 18 O2: 100%
Worsening	Recognize deterioration in mental status and signs of increased ICP. Order mannitol or hypertonic saline and elevate head of bed to 30 degrees.	Tachypnea, left mildly dilated and sluggishly reactive pupil, GCS: 8 E-2 V-2 M-4, coarse breath sounds. If the learner does not order mannitol or hypertonic saline, the nurse states, “He’s not looking so good,” and HR to 20, BP 183/92, RR 30, O2 89% Otherwise, proceed to intubation preparation.	T: 37.0° C HR: 50 BP: 170/60 RR: 30 O2: 89%
Intubation Preparation	Prepare rapid sequence intubation (RSI) Re-evaluate neurologic exam. Consider using succinyl-choline given shorter duration.	Patient remains unresponsive with coarse breath sounds. If not recognizing the need for intubation, the patient will start making gagging noises. If the team does not verbalize medications, nurse prompts, “What medications do you want for RSI, doctor?”	T: 37.0° C HR: 60 BP:160/100 RR: 22 O2:100% BVM
Intubation	Intubate patient. Confirm endotracheal tube (ETT) placement.	If intubated properly, **display post-intubation x-ray**. Proceed to next state. If no breath sounds or mannikin software indicates esophageal intubation, oxygen saturation drops to 80%. Allow learner to re-attempt intubation. When successful, proceed to next state.	T: 37.0°C HR: 60 BP:160/100 RR: 22 O2:100% BVM
Post-intubation Care	Discuss ventilator settings with consideration for increased RR.	If no ventilator settings are given, nurse prompts, “Respiratory Therapy wants to know what settings you want.”	T: 37.0°C HR: 60, BP: 160/100 RR: 22 O2: 100% ETT
Disposition	Contact neurosurgery consultant and arrange transfer.	Neurosurgeon embedded participant will respond after the patient is presented. After the presentation, respond, “We are happy to accept the patient in transfer.” If the presentation did not discuss shunt tap, ask, “Did you tap the shunt?” Then respond with, “You should not need to perform the shunt tap at this time. Shunts can only be tapped so many times, and it does not sound like that is needed now.” End of case. For advanced learners, can proceed to optional shunt tap step. If the learner does not ask for neurosurgery, nurse will prompt, “Do you want me to call anyone for you?”	T: 37.0°C HR: 80 BP: 120/90 RR: 22 O2: 100% ETT
Optional: Shunt Tap	Recognize sudden deterioration. Call neurosurgeon.	While arranging for transfer, patient’s vital signs worsen. GCS 3T. Paralyzed Advance to next state when learner asks for neurosurgeon. If no call is placed to the neurosurgeon, nurse or mother can ask, “What is going on? Is he getting worse? Is there anything else we can do? Can we get the fluid out of him?”	T: 37.0°C HR: 30 BP: 180/100 RR: 22 O2: 100% ETT
Shunt Tap Prep	Discuss shunt tap with neurosurgeon.	Learners provide update on patient status. Neurosurgeon will ask, “So what do you think is going on here?” Then they say, “It sounds like the patient is having increased ICP and might be herniating.” If the learner asks about performing the shunt tap, neurosurgeon will state, “I agree with the shunt tap. Please perform this and we will work on expediting transfer.” Proceed to next state. If the learner does not ask about performing the shunt tap, the neurosurgeon should say, “I would suggest trying to stabilize the patient a little more before transfer. You should perform an emergency shunt tap to decompress the pressure a little before transfer.” Proceed to next state.	T: 37.0°C HR: 30 BP: 180/100 RR: 22 O2: 100% ETT
Shunt Tap Performed	Perform shunt tap or discuss steps. Successful shunt ends case.	Learners perform the steps for a shunt tap or verbalize steps. When learner completes procedure, HR 63, BP 129/87, RR 18, O2: 100% End case.	T: 37.0°C HR: 30, BP: 180/100 RR: 22 O2: 100% ETT

**Table:** 

**Critical Actions:** Evaluate airway, breathing, and circulation. Obtain history and perform thorough exam including evaluation of shunt. Order Shunt series and CT scan of the head. Intubate patient for airway protection. Treat increased intracranial pressure with hyperventilation, elevation of the head of bed, 3%saline or mannitol. Consult neurosurgery.	0:00

**Table:** 

	Milestone	Did not achievelevel 1	Level 1	Level 2	Level 3
1	**Emergency Stabilization (PC1)**	□ Did not achieve Level 1	□ Recognizes abnormal vital signs	□ Recognizes an unstable patient, requiring intervention Performs primary assessment Discerns data to formulate a diagnostic impression/plan	□ Manages and prioritizes critical actions in a critically ill patient Reassesses after implementing a stabilizing intervention
2	**Performance of focused history and physical (PC2)**	□ Did not achieve Level 1	□ Performs a reliable, comprehensive history and physical exam	□ Performs and communicates a focused history and physical exam based on chief complaint and urgent issues	□ Prioritizes essential components of history and physical exam given dynamic circumstances
3	**Diagnostic studies (PC3)**	□ Did not achieve Level 1	□ Determines the necessity of diagnostic studies	□ Orders appropriate diagnostic studies. Performs appropriate bedside diagnostic studies/procedures	□ Prioritizes essential testing Interprets results of diagnostic studies Reviews risks, benefits, contraindications, and alternatives to a diagnostic study or procedure
4	**Diagnosis (PC4)**	□ Did not achieve Level 1	□ Considers a list of potential diagnoses	□ Considers an appropriate list of potential diagnosis May or may not make correct diagnosis	□ Makes the appropriate diagnosis Considers other potential diagnoses, avoiding premature closure
5	**Pharmacotherapy (PC5)**	□ Did not achieve Level 1	□ Asks patient for drug allergies	□ Selects an medication for therapeutic intervention, consider potential adverse effects	□ Selects the most appropriate medication and understands mechanism of action, effect, and potential side effects Considers and recognizes drug-drug interactions
6	**Observation and reassessment (PC6)**	□ Did not achieve Level 1	□ Reevaluates patient at least one time during case	□ Reevaluates patient after most therapeutic interventions	□ Consistently evaluates the effectiveness of therapies at appropriate intervals
7	**Disposition (PC7)**	□ Did not achieve Level 1	□ Appropriately selects whether to admit or discharge the patient	□ Appropriately selects whether to admit or discharge Involves the expertise of some of the appropriate specialists	□ Educates the patient appropriately about their disposition Assigns patient to an appropriate level of care (ICU/Tele/Floor) Involves expertise of all appropriate specialists
9	**General Approach to Procedures (PC9)**	□ Did not achieve Level 1	□ Identifies pertinent anatomy and physiology for a procedure Uses appropriate Universal Precautions	□ Obtains informed consent Knows indications, contraindications, anatomic landmarks, equipment, anesthetic and procedural technique, and potential complications for common ED procedures	□ Determines a back-up strategy if initial attempts are unsuccessful Correctly interprets results of diagnostic procedure
20	**Professional Values (PROF1)**	□ Did not achieve Level 1	□ Demonstrates caring, honest behavior	□ Exhibits compassion, respect, sensitivity and responsiveness	□ Develops alternative care plans when patients’ personal beliefs and decisions preclude standard care
22	**Patient centered communication (ICS1)**	□ Did not achieve level 1	□ Establishes rapport and demonstrates empathy to patient (and family) Listens effectively	□ Elicits patient’s reason for seeking health care	□ Manages patient expectations in a manner that minimizes potential for stress, conflict, and misunderstanding. Effectively communicates with vulnerable populations, (at risk patients and families)
23	**Team management (ICS2)**	□ Did not achieve level 1	□ Recognizes other members of the patient care team during case (nurse, techs)	□ Communicates pertinent information to other healthcare colleagues	□ Communicates a clear, succinct, and appropriate handoff with specialists and other colleagues Communicates effectively with ancillary staff

**Table 1:** Results from the post simulation survey. n=11

Survey item	1 n (%)	2 n (%)	3 n (%)	4 n (%)	5 n (%)	Median
Confidence recognizing signs and symptoms of VP shunt dysfunction	0	2 (18.2)	1 (9.1)	6 (54.5)	2 (18.2)	4
Confidence managing complications of VP shunt dysfunction	0	2 (18.2)	2 (18.2)	5 (45.5)	2 (18.2)	4
Previous experience with VP shunt dysfunction	6 (54.6)	3 (27.3)	1 (9.1)	0	1 (9.1)	1
Finding this simulation helpful to my future practice as an emergency physician	0	0	0	2 (18.2)	9 (81.8)	5

**Figure:**
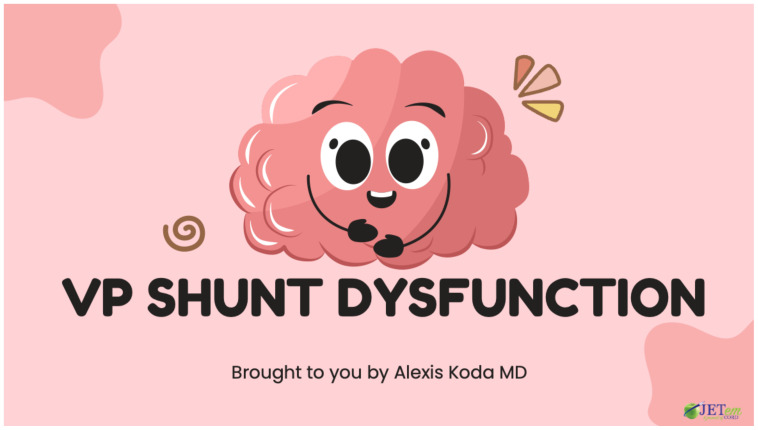
Please see associated PowerPoint file

## INSTRUCTOR MATERIALS

**Case Title:** VP Shunt Malfunction

**Case Description & Diagnosis (short synopsis):** A 9-year-old male with a history of hydrocephalus and VP shunt presents with altered mental status, headache, and vomiting. The patient demonstrates signs of increased intracranial pressure (ICP) due to a fractured VP shunt. The case progresses through initial assessment, clinical deterioration, intubation, and consultation with neurosurgery for definitive management. An optional shunt tap can be performed.

**Equipment or Props Needed:**

- High-fidelity pediatric mannequin
- Cardiac monitor with defibrillation, pacing, and monitoring abilities
- IV access supplies
- Airway equipment (bag-valve mask, laryngoscope, endotracheal tube (ETT), stylet, CO2 detector)
- Supplemental O2 devices (nasal cannula, non-rebreather mask)
- Mock IV fluids (labeled hypertonic saline, normal saline, lactated Ringers, mannitol)
- Mock Rapid Sequence Intubation (RSI) medications (Etomidate, Succinylcholine, Rocuronium, Propofol)
- Custom VP shunt reservoir model (as described below)
- Equipment for VP Shunt Tap:
  ○ Povidone iodine solution or chlorhexidine swab
  ○ local anesthetic
  ○ 25-gauge butterfly needle
  ○ 3-way stop cock
  ○ Manometer from lumbar puncture kit and collection tubes if needed

**Embedded participants needed:**

Invited participants to play mother and nurse. Faculty member to call in as the neurosurgeon consultant.

**Stimulus Inventory:**

**Background and brief information:** The patient is a 9-year-old male brought in by ambulance to a community hospital with basic services including general surgery and anesthesia. There are no specialized services such as neurosurgery or pediatric specialists. Any specialists at other hospitals may be contacted and consulted to arrange for transfer; however, they are not available to do procedures. Learners should be pre-briefed on the case setting. The mother reports altered mental status.

**Initial presentation:** A 9-year-old male presents with altered mental status. The mother reports that the child complained of a headache yesterday. Today, she states he is very drowsy and “not acting like himself.” He had one episode of non-bloody, non-bilious vomiting. The child is able to provide minimal history, stating only that his “tummy doesn’t feel good” and “my head hurts.”

**How the scenario unfolds:** The patient arrives for initial evaluation, where the learners must conduct a thorough history and physical examination with particular attention to the ventriculoperitoneal shunt. The learner will need to order the appropriate initial imaging studies and labs in order to diagnose the VP shunt dysfunction. As the scenario unfolds, the patient’s condition deteriorates, showing classic signs of increased intracranial pressure (bradycardia, hypertension, and declining mental status). The medical team must quickly recognize these warning signs and initiate appropriate interventions for ICP management, including preparation for intubation. The learners must stabilize the patient, consult the neurosurgeon, and arrange transfer to a pediatric hospital with neurosurgical capabilities.

The scenario can be adapted for advanced learners. If desired, the learners can be directed to perform a shunt tap after a discussion with the neurosurgeon. If this is desired, the patient further deteriorates despite providing care for increased intracranial pressure.

**Critical actions:**

1. Evaluate airway, breathing, and circulation
2. Obtain history and perform thorough exam including evaluation of shunt
3. Order shunt series and CT scan of the head
4. Intubate patient for airway protection
5. Treat increased intracranial pressure with hyperventilation, elevation of the head of bed, 3% saline or mannitol
6. Consult neurosurgery

**Case Title:** VP Shunt Malfunction

**Chief Complaint:** Altered mental status, headache, vomiting

**General Appearance:**

**Primary Survey:**

- **Airway:** Intact, able to answer some questions
- **Breathing:** Clear breath sounds, tachypneic
- **Circulation:** Brisk capillary refill, 2+ pulses throughout, bradycardic

**History:**

- **History of present illness:** The patient is a 9-year-old male with a history of “water on the brain.” The patient has had one day of headache and is more drowsy and confused than usual. He is also having nausea with one episode of non-bloody non-bilious vomiting. No noted fever, runny nose, sore throat, coughing, diarrhea. No shaking or loss of consciousness.
- **Past medical history:** History of “water on the brain”; up to date on vaccines.
- **Past surgical history:** VP shunt placement 1 year ago
- **Patient’s medications:** None
- **Allergies:** None
- **Social history:** Lives with parents
- **Family history:** Non-contributory

**Secondary Survey/Physical Examination:**

- **General appearance:** Ill-appearing child, lethargic
- **HEENT:**
  ○ **Head:** Atraumatic, normocephalic except for shunt on R parietal scalp. When checking the child’s VP shunt reservoir, if the reservoir is compressed, it is tense
  ○ **Eyes:** Sluggish pupils, otherwise normal
  ○ **Ears:** Normal
  ○ **Nose:** Normal
  ○ **Throat:** Normal
- **Neck:** Normal
- **Heart:** Bradycardia, no murmurs, gallops, or clicks
- **Lungs:** Clear to auscultation, tachypneic
- **Abdominal/GI:** Soft, mildly tender diffusely
- **Genitourinary:** Normal
- **Rectal:** Normal
- **Extremities:** Normal
- **Back:** Normal
- **Neuro:** Some confusion and slow answers. Increasingly lethargic. Moving all extremities with 5/5 strength. Sensation intact. No difficulty with finger to nose and heel to shin.
- **Skin:** Normal
- **Lymph:** Normal
- **Psych:** Normal

*Abdominal X-Ray*

Sheikh, Y. Fractured VP Shunt: Case study. In: Radiopaedia. https://radiopaedia.org/cases/fractured-vp-shunt?lang=us. Published September 12, 2021. Accessed March 19, 2025. CC BY-NC SA 3.0.

*Head CT*

Bickle, I. Acute hydrocephalus from VP shunt blockage. In: Radiopedia. https://radiopaedia.org/cases/55488. Published September 9, 2017. Accessed March 19, 2025. License CC BY-NC-SA 3.0.

*Post-intubation X-Ray*

Heilman J. ETtubeGoodPositionNo.png. In: Wikimedia Commons. https://commons.wikimedia.org/wiki/File%3AETtubeGoodPositionNo.png. Published May 28, 2016. Accessed March 19, 2025. License CC BY-SA 4.0

## OPERATOR MATERIALS

**Diagnosis:** Ventriculoperitoneal shunt dysfunction from shunt fracture.

**Disposition:** Transfer to a neurocritical care unit at a pediatric hospital.

## DEBRIEFING AND EVALUATION PEARLS

### VP Shunt Malfunction

#### Signs and Symptoms of Shunt Dysfunction^4^

- Headache
- Lethargy/Mental Status Change/Coma
- Decreased intellectual performance
- Ataxia
- Nausea/Vomiting
- Abdominal pain
- Autonomic instability
- Rarely seizures
- Cushing’s triad (hypertension, bradycardia, irregular breathing) indicates significantly increased ICP^7^,^8^

#### Types of Shunt Dysfunction^6^

- Infection
  ○ Highest risk in first 6 months
  ○ Staphylococcus epidermidis or aureus
  ○ After first 6 months, consider migration from the abdomen and (rarer) fungal infections
- Mechanical
  ○ Blockage (ie, abscess), kinking or breakage, incorrect positioning/migration
  ○ Most common causes
    ■ Adhesions
    ■ Kinking
    ■ Infection
    ■ CONSTIPATION!!!
  ○ Most common in 1st year after placement
  ○ Most common proximal cause: the growth of choroid plexus or ependymal tissue
  ○ Most common distal cause: constipation or migration (normal growth of child)
  ○ Fracture = 2nd most common
- Functional
  ○ Over-draining
  ○ Under-draining
  ○ Ventricular slit syndrome^7^
    ■ Small ventricles on imaging
    ■ Headache worse in upright; better lying flat=opposite of normal obstructive process

#### Causes of Shunt Dysfunction

- Intraventricular hemorrhage
- Constipation
- Brain tumors
- Malpositioning
- Congenital Myelomeningocele
- Knot/Kinking
- Distal Migration
- Adhesion
- Choroid plexus or ependymal growth
- Shunt perforation
- Slit ventricle syndrome
- Abdominal pseudocyst formation

#### Risk Factors for Shunt Dysfunction

- Time from 1st placement
- Time from 1st revision
- Young age
- Number of VP shunts in place
- Race
  ○ Increased risk in Hispanic, African American, and Native American populations

#### Treatment of Increased ICP

- Supportive Management
  ○ ABCs
- Treatment of ICP
  ○ Hypertonic saline vs. mannitol
  ○ Elevate head of bed (HOB)
  ○ Hyperventilation
  ○ Shunt tap
- Treatment of the cause of malfunction
  ○ Antibiotics
  ○ Surgery

#### Pearls

- Ventriculoperitoneal shunt complications occur at a rate of up to 5% of patients per year.^9^
- The shunt reservoir can provide valuable diagnostic information; if compressed, it should refill; failure to refill suggests obstruction.
- Consider shunt tap only after discussion with neurosurgery.^2^,^5^ Shunts can only be tapped so many times. Usually, neurosurgery performs the shunt to send fluid for culture and to rule out infection. However, shunt taps can be therapeutic as well. They are usually done by the neurosurgeon. However, it is something that we may be asked to do and should be familiar with doing.

#### Ventriculoperitoneal Shunt Tap^2^,^5^

1. Obtain informed consent.
2. Identify the shunt reservoir by palpating the prominence on the posterior aspect of the patient’s skull (usually on the right).
3. Prepare a sterile 25-gauge butterfly needle. Obtain a 3-way stopcock and manometer and sample tubes from a lumbar puncture kit if pressure needs to be measured.
4. Place the patient in the lateral decubitus position with the shunt reservoir facing up.
5. Prep the site with chlorhexidine or iodine. Place sterile drapes.
6. Obtain a spinal needle and attach to the syringe. If measuring pressure, attach tubing from a 25-G needle and connect to the 3-way stopcock and manometer using sterile technique.
7. With the manometer at the level of the patient’s ear, insert the needle into the skin and advance the needle into the shunt and observe for cerebrospinal fluid (CSF) flow. Stop advancing once fluid is obtained. If not measuring pressure, perform the same steps with the needle and attached syringe.
  - A pressure of greater than 25 mmHg indicates a strong likelihood for distal obstruction.
  - Remove fluid until pressure is less than 15 mmHg.
  - Aspirate slowly to prevent intracranial bleeding.
  - Decreased flow indicates proximal obstruction. Consult neurosurgery.
8. Withdraw needle and hold pressure with sterile gauze for 2 minutes and apply sterile dressing.

#### Other debriefing points

- Ask learners what made them suspect VP shunt malfunction.
- Discuss their approach to the physical examination of a VP shunt.
- Review their decision-making regarding imaging studies.
- Discuss concerns about radiation exposure in pediatric patients versus clinical necessity.
- Review indications and contraindications for shunt tap.
- Discuss transfer considerations for a community hospital managing a pediatric neurosurgical emergency.

**Wrap Up:** See Appendix A: VP Shunt Dysfunction PowerPoint presentation.

## SIMULATION ASSESSMENT

*VP Shunt Malfunction*

Learner: _________________________________________

***Assessment Timeline***

This timeline is to help observers assess their learners. It allows observer to make notes on when learners performed various tasks, which can help guide debriefing discussion.

**Critical Actions:**

□ Evaluate airway, breathing, and circulation.
□ Obtain history and perform thorough exam including evaluation of shunt.
□ Order Shunt series and CT scan of the head.
□ Intubate patient for airway protection.
□ Treat increased intracranial pressure with hyperventilation, elevation of the head of bed, 3% saline or mannitol.
□ Consult neurosurgery.

**Summative and formative comments:**

**Milestones assessment:**
